# Chronic hepatitis B in Korean Americans: decreased prevalence and poor linkage to care

**DOI:** 10.1186/s12879-016-1732-7

**Published:** 2016-08-15

**Authors:** Chul S. Hyun, Sue Kim, Seung Y. Kang, Seo Jung, Seulgi Lee

**Affiliations:** 1 Holy Name Medical Center, Teaneck, NJ USA; 2 Center for Viral Hepatitis, 35 Van Nostrand Avenue, Englewood, NJ 07631 USA

**Keywords:** Hepatitis B screening, Korean Americans, Linkage to care

## Abstract

**Background:**

Chronic hepatitis B virus(HBV) infection is a major cause of liver related morbidity and mortality. HBV infection remains largely underdiagnosed in Asian American population, and it is also poorly linked to clinical care. We, therefore, assessed the HBV prevalence and evaluated linkage to care among Korean Americans in order to develop strategic plans to reduce the impact of HBV in a high risk community.

**Methods:**

Serologic screening and survey were provided to 7157 Korean American adults (age 21–100) in New Jersey between December 2009 and June 2015. All participants were tested for hepatitis B surface antigen (HBsAg), hepatitis B surface antibody (anti-HBs), and hepatitis B core IgG antibody (anti-HBc). Additional survey was conducted on the subjects chronically infected with HBV on their history of infection. Self-administered questionnaires were employed to evaluate demographic and epidemiologic characteristics.

**Results:**

Of those 7157screened, 171 (2.4 %) were HBV infected, 2736(38.2 %) were susceptible to HBV, and 4250(59.4 %) were immune. The prevalence of chronic HBV varied between the age groups: 1.18 % (age21-30); 2.53 % (age 31–40); 2.76 % (age 41–50); 2.90 % (age 51–60); 2.06 % (age 61–70); and 1.37 % (age 71–100). The rate of HBsAg was significantly higher in males (3.04 %) as compared to females (1.93 %). At least 75 % of these HBV infected subjects had been previously diagnosed, but were not engaged in care.

**Conclusion:**

This screening study suggests that the HBV prevalence in Korean Americans is significantly lower than currently understood. On the other hand, many of the individuals chronically infected with HBV cannot access care, suggesting a poor linkage-to-care (LTC). Further, a large percentage of the population is still susceptible to HBV. Study findings will be used to develop strategies to tailor community-based HBV screenings and LTC to the high risk populations.

## Background

Chronic hepatitis B (CHB) is a serious global health issue affecting more than 240 million people. Approximately 25 % or more of these CHB patients may eventually suffer from liver cirrhosis, primary liver cancer or other complications of CHB [1–4]. There are three major issues regarding CHB today. First, numerous people have not been screened and thus are not aware of the fact that they are infected [5, 6]. Second, despite the availability of vaccine since early 1980s, many are still at risk and require vaccination [6, 7]. Third, even with the availability of effective antiviral treatment for CHB, a majority of the CHB treatment candidates are not linked to adequate care [8–11].

There is a marked disparity between different racial and ethnic groups in the prevalence of CHB and its complications. For instance, 5–10 % of all Asian Americans have HBV infection as compared with 0.2 % of Caucasian Americans [3, 5]. In the United States alone, an estimated two million people are chronically infected with HBV. Nearly 70 % of these HBV infected individuals are unaware they have the virus. Furthermore, less than 10% of patients who may need treatment in the United States are currently receiving antiviral medication. Thus, hepatitis B is not only significantly underdiagnosed, but it is also undertreated [6, 7].

The fact that the majority of the people chronically infected with HBV are not aware of their infection clearly demonstrates that there are barriers to screening in the United States. The barriers to screening are multi-factorial. Patient-related obstacles are mostly consisted of lack of awareness about the disease, language and cultural barriers, and financial issues [8, 12]. Additionally, providers and healthcare system currently available in the US lack the understanding of the significance of CHB. To be specific, there is a lack of public health systems to meet the needs of multicultural populations [9, 13]. There is also a poor communication between providers and patients of different racial, ethnic, or cultural backgrounds. Last but not least, there is a significant lack of cross-cultural training in health professionals [11, 14, 15]. As a result, there is a serious lack of adequate health access models available for minority populations.

New Jersey and its vicinity are heavily concentrated with Asian Americans, many of whom are currently infected with HBV. Despite a rapidly growing population of Korean Americans in New Jersey, a majority of these people have not been accessed by the currently available HBV screening program. During the period between December 2009 and June 2015, Center for Viral Hepatitis (CVH) and Asian Liver Center (ALC) of Holy Name Medical Center carried out a total of 128 community outreach HBV screening events in Central and Northern New Jersey. Serologic screening and survey were provided to a total of 7199 Korean American adults (mean age 52) throughout these events. All the participants were tested for hepatitis B surface antigen (HBsAg), hepatitis B surface antibody (anti-HBs), and hepatitis B core IgG antibody (anti-HBc).

This study and the results of other recent studies clearly suggest that the reported prevalence of hepatitis B among Koreans in both Korea and the United States may be higher than the true prevalence [16, 17]. The results of the current study also indicate an urgent need to improve vaccination and LTC to prevent HBV- related liver disease and cancer.

## Methods

### Study design

A large scale community-based Hepatitis B Screening and Awareness Campaign was led by CVH and ALC of Holy Name Medical Center in the state of New Jersey. CVH and ALC are two major non-profit organizations devoted to promoting CHB screening and linkage to care in Asian American community. All our staff were consisted of community physicians with expertise in the field of CHB, nurses, and community volunteers. CVH and ALC have collaborative relationship with local hospitals, community organizations and physicians to work cooperatively in a multidisciplinary manner to ensure the highest quality of care for screening, education, vaccination, and linkage to care for the individuals chronically infected with HBV.

Our hepatitis B campaign consisted of a total of 128 community outreach screenings and education seminars at various locations throughout Central and Northern New Jersey during the period between December 2009 and June 2015. CVH and ALC organized and hosted all the campaign events. A total of 7199 Korean American immigrants were screened for hepatitis B in 98 churches, 12 community centers, and 18 health fairs in five different counties of New Jersey. All the HBV infected subjects were referred to specialists within the community, and those who were not immune to HBV were vaccinated.

### Participant characteristics

All the participants were Korean Americans currently residing in New Jersey. 99.5 % of the participants were born in Korea. Their ages ranged from 21 to 100, with a mean age of 52. A vast majority (>85 %) of the participants were reported to having lived in the United States for a minimum of 10 years. More than 99 % of the participants preferred Korean as the language for communication.

### Serological screening and survey

Hepatitis B Screening included the three following tests: HBsAg, anti-HBs, and anti-HBc (IgG). The blood drawing and processing were conducted by phlebotomists or registered nurses, and all the results were reviewed by physicians. All the participants who had screening tests were contacted by the following means of communication: telephone and mail. The participants who did not respond, for instance, were tracked down and were persistently contacted through the means of telephone calls and certified mails.

Self-administered questionnaires were employed to evaluate demographic and epidemiologic characteristics. At the time of the screening, participants completed a survey written in both English and Korean. The items in the survey included gender, date of birth, country of birth, years in the United States, and preferred language of communication. All participants provided written informed consent. Some of the screening events also included Hepatitis B awareness seminars and question and answer sessions to address numerous questions the participants had.

### Data analysis

Exact binomial 95 % confidence intervals (CIs) were calculated for prevalence of HBsAg seropositivity. A 2-tailed Fisher's exact test was used to test for differences between frequencies.

## Results

### Sample demographics

**Fig. 1:**
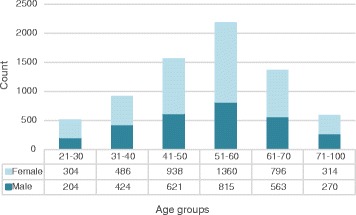
Distribution of the study sample by age and gender

We conducted a total of 128 screening events in Central and Northern New Jersey between December 2009 and June 2015. Figure 1 shows the age and gender distribution of 7095 participants. Of 7199 screened, 104 were unspecified. The average age was 52 years, and 58.3 % were females. Approximately half of the participants belonged to the age group of 41–60.

### Serological data

**Fig. 2:**
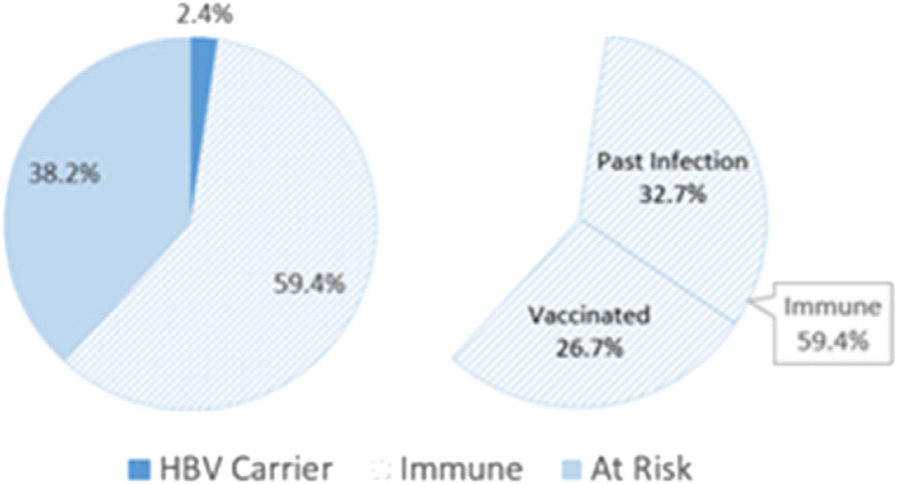
Hepatitis B status in a Korean American population in New Jersey

Of 7199 Korean Americans screened, the results for 7157 participants were available. Of 7157 participants, 171 (2.4 %) were HBV infected, 4250 (59.4 %) were immune, and 2736 (38.2 %) were susceptible to HBV (Fig. 2). Of 4250 (59.4 %) immune subjects, 2319 (32.7 %) were recovered from past infection, and 1931 (26.7 %) were vaccinated.

The prevalence of HBV varied significantly between age groups: 1.18 % (age21-30); 2.53 % (age 31–40); 2.76 % (age 41–50); 2.90 % (age 51–60); 2.06 % (age 61–70); and 1.37 % (age 71–100). The highest HBsAg positive rates are found in the group between age 41 and 60. To determine a statistical difference between age groups, we compared the HBV prevalence in the youngest age group (age 21–30) with older age groups. The HBV prevalence in the age group of 21–30 years was significantly different only from two other age groups (age 41–50 and age 51–60) with the p-values of 0.0436 and 0.028, respectively (Table 1).

**Table 1:** Prevalence of HBsAg seropositivity by age

Age groups	Participant number	HBsAG seropositive participants	HBSAg seropositivity	95 % CI (%)
21 - 30	508	6	1.18	0.43-2.55
31 - 40	910	23	2.53	1.61-3.77
41 - 50	1559	43	2.76	2.00-3.70
51 - 60	2175	63	2.90	2.23-3.69
61 - 70	1359	28	2.06	1.37-2.96
71 - 100	584	8	1.37	0.59-2.68

**Fig. 3:**
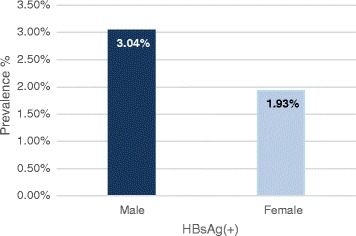
The prevalence rate by gender

Of 171 HBV infected subjects, all but three were born in Korea. There were 88 males, 81 females, and 2 unknown. As shown in Fig. 3, the rate of HBsAg is significantly higher in males (3.04 %) as compared to females (1.93 %) (*p* = 0.003).

### Infection history of the HBsAg-positive subjects

**Fig. 4:**
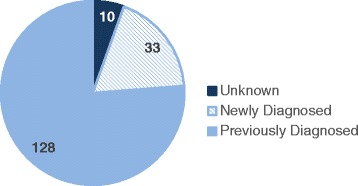
History of HBV infection in the HBsAg-positive subjects

A survey was carried out to determine how many of these HBsAg-positive subjects might have known their infection status prior to the current screening (Fig. 4). It showed that out of 171 HBV infected subjects, 128 knew that they were chronically infected prior to the screening, 33 just learned that they were infected, and the remaining 10 were not sure of their hepatitis B status. Of those 128 previously diagnosed subjects, at least 105 have reported of having immigrated to the United States before 2000. About 10 % of these HBV infected subjects have seen physicians for hepatitis B, but none of them engaged in any further work up. A vast majority (>95 %) of these subjects had no symptoms or were not aware of potential complications of hepatitis B such as cirrhosis and liver cancer.

### Acquisition of immunity

**Fig. 5:**
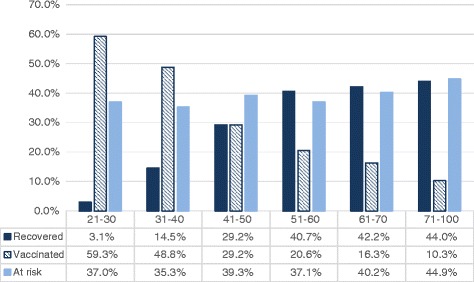
Acquisition of immunity in all age groups. The percentage values represent percent of participants in each age group

Of 7157 participants screened, a total of 4250 were immune, showing 59.4 % immune rate (Fig. 2). We looked into acquisition of immunity from vaccination versus past infection. Of 4250 immune participants, 1931 participants developed immunity from vaccination, and the remaining 2319 participants developed immunity from past infection. The ratio of the acquisition of immunity from vaccination to the acquisition of immunity from past infection varied in different age groups. As shown in Fig. 5, the majority of immunity came from vaccination in younger age groups (age 21–40) while the majority of immunity came from previous infection in older age groups (51–100).

### Lack of immunity in all age groups

Despite the availability of vaccine for over three decades, there were still many subjects who were not immune to HBV. Of 7157 screened in this study, 2736 participants (38.2 %) lacked protective antibody to HBV (Fig. 2). This lack of immunity was apparent in all age groups (Fig. 5).

## Discussion

In the current study of our community-based hepatitis B screening campaign, we performed an epidemiologic evaluation on hepatitis B in Korean Americans. On a total of 7157 participants screened between December 2009 and June 2015 from New Jersey, this study shows that the prevalence of CHB in Korean American adults is 2.4 %, which is significantly lower than currently understood [16, 17]. Surprisingly, a majority of the HBV infected subjects (80.7 %) had not been linked to adequate care for monitoring and treatment. In addition, we found 59.4 % as immune and 38.2 % as susceptible to HBV infection.

One noteworthy aspect of this study is the demonstration of a HBV prevalence of 2.4 % among Korean American immigrants, which is substantially lower than currently believed. The prevalence of chronic hepatitis B infection for Koreans in South Korea has been also reported to be as high as 8 % until recent years [16, 17]. In a review of the studies on the Korean American population, we find a trend of decreasing prevalence rates of hepatitis B over the past three decades. A community-based Hepatitis B screening campaign by Hann et al. have evaluated 6130 Korean Americans in eastern United States between 1988 and 1990 and showed a HBsAg positive rate of 6.1 %, with 8 % for males and 4.4 % for females [18]. Another community- based study on 609 Korean Americans in Colorado between 2004 and 2007 revealed a HBV prevalence of 4 % [19]. It also revealed 55 % immune and 41 % susceptible to infection. More recently, a study on 973 Korean American residents from California, which took place between 2009 and 2010, demonstrated a 3.0 % HBV prevalence rate [20]. In this study, the immune rate was higher at 76 %, and only 20 % were susceptible to infection.

The relatively low HBV prevalence found in the current study is congruent with the results of the recent studies from Korea. In an evaluation of 50,140 Korean participants for HBsAg positivity over a period of 12 years between 1998 and 2010, investigators found a decreasing rate of HBsAg positivity: 4.61 % in 1998; 3.69 % in 2005, and 2.98 % in 2010 [21]. They also showed that the percentage of HBV infected individuals in the age group of 10–20 years decreased from 2.2 % in 1998 to 0.12 % in 2010. In addition, the percentage of the HBV infected in the age group 10–39 years decreased from 4.72 % in 1998 to 2.29 % in 2010. According to these studies, South Korea may belong to low intermediate endemic (2-4 %) area as recently reported by CDC Health Information for International Travel 2016 [22].

The decreasing HBV prevalence in Koreans in the United States and Korea may be largely attributed to HBV immunization programs and other preventive strategies [23]. Korean national Immunization Program for all neonates began in 1995 to include universal vaccination, regardless of maternal HBsAg status, which helped to prevent both vertical and horizontal transmission [23, 24]. Likewise, universal vaccination programs have contributed to the marked decline in HBV prevalence rate in people younger than 20 years of age worldwide [18, 23]. A similar decline in HBV prevalence was reported in Taiwan following its neonatal vaccination program [25]. These are congruent with results of the current study, which show an increased vaccination rate and a decreased HBV prevalence in younger population.

There are important limitations to consider in this study. First, the participants, namely Korean American residents in New Jersey may not be representative of the overall Korean population in the United States. Education level and socioeconomic status, for instance, are important factors known to affect HBV prevalence [26, 27]. How these factors might have influenced the results of the current study are unknown. Secondly, the age distribution in our study population may not represent the entire population. For instance, our study sample under-represents younger age groups relative to the older age groups. Youngest age group below 20, who are known to have a very low HBV prevalence, for instance, are not represented in our study population. Thirdly, it is difficult to determine how the screening rates would have affected the prevalence. For instance, many of our study subjects underwent screening knowing their hepatitis B status. If a greater number of HBV infected subjects in proportion to non-infected subjects underwent the screening in this campaign, the HBV prevalence would have been overestimated. Finally, differences in the settings for screening could have affected the prevalence. It should be noted, however, that the non-clinical community settings for screening employed in this study were similar to the community settings for screening employed in previous studies which had demonstrated significantly higher HBV prevalence [18, 19].

Although the HBV prevalence is significantly declined, the percentage of the Korean American population susceptible to HBV infection remains high. More than one third of the participants were susceptible, and this lack of immunity was evident throughout all the age groups (Fig. 5). It is also remarkable to note that a substantial percentage of a younger age group (age 21–30) still remains susceptible to HBV infection. In this age group, approximately 3.1 % had hepatitis B infection previously, and 59.3 % had detectable anti-HBs level as result of vaccination. Since 5-10 % of the persons vaccinated against hepatitis B may not respond [28], we can estimate that approximately one third of the subjects in this age group are not vaccinated.

Another noteworthy aspect of this study is the demonstration that a majority of the HBV infected subjects found in the current campaign had been previously diagnosed (Fig. 4). These individuals chronically infected with HBV are not engaged in any care, reflecting a poor hepatitis B LTC in Korean American community. Other investigators have also reported that only a minority of HBV infected people can access care [9-11]. In fact, it was estimated that only 40 % of the HBV infected subjects screened in community setting were successfully linked to care [10, 11]. These challenges met in LTC are most often related to finding qualified providers within the community, who can provide care in a linguistically and culturally sensitive manner. Improvements in patient education, counseling, and navigation efforts may also be an effective strategy to improve linkages from community-based testing sites to HBV-directed medical care. Because routine and ongoing monitoring is the foundation for an effective HBV medical management, future efforts to improve outcomes among HBV infected people should provide a greater emphasis on LTC.

This study illustrates how Community-based screening can provide an effective screening for CHB in ethnic minority populations, where language, culture, and financial barriers may preclude them to access appropriate care. There are more than several ways community-based health initiatives can be achieved, depending upon who initiates, drives and carries out these initiatives and the degree of their input [29, 30]. In the current community-based campaign for hepatitis B, individual leaderships and community organizations carried out the entire project with an external fiscal support. We offered comprehensive services including screening programs, educational outreach, vaccination, and a link to community physicians with expertise in hepatitis B care. Of note, our campaign also included education seminars and forums for physicians who had greatest contacts with the Asian American community. In addition to updating physicians on the effective HBV prevention, antiviral treatments and monitoring, these educational activities helped motivate providers to get involved in community outreach. Secondly, we fostered strategic partnerships with key community organizations which promoted community ownership and sustained engagement of the campaign. As a result, a total of 160 subjects chronically infected with HBV identified in this campaign were referred to specialists for confirmatory testing, monitoring, and antiviral treatments. Among these HBV infected subjects referred to specialists, 76 % had initial consultations within a 6 month period.

## Conclusion

The current study on one of the largest HBV screening campaigns among Korean Americans demonstrates a significantly decreased HBV prevalence of 2.4 %. The study also reveals a poor LTC for those HBV infected subjects, who require monitoring and/or medical treatments. A comprehensive, community-based screening and evaluation program described in this report may be effectively implemented in other ethnic populations to facilitate hepatitis B care.

## Abbreviations

anti-HBc, hepatitis B core IgG antibody; anti-HBs, hepatitis B surface antibody; CHB, chronic hepatitis B; HBsAg, hepatitis B surface antigen; HBV, hepatitis B virus; LTC, linkage to care.
